# A TAVI Programme Without an On-Site Cardiac Surgery Department: A Single-Center Retrospective Study

**DOI:** 10.3390/jcm14155449

**Published:** 2025-08-02

**Authors:** Rami Barashi, Mustafa Gabarin, Ziad Arow, Ranin Hilu, Ilya Losin, Ivan Novikov, Karam Abd El Hai, Yoav Arnson, Yoram Neuman, Koby Pesis, Ziyad Jebara, David Pereg, Edward Koifman, Abid Assali, Hana Vaknin-Assa

**Affiliations:** 1 Department of Cardiology, Meir Medical Center, Kfar Saba 4428164, Israel; mustafa.gabarin88@gmail.com (M.G.); ziad.arow@gmail.com (Z.A.); raneen.hellou@gmail.com (R.H.); ilyalosin@gmail.com (I.L.); dr.novikov.ia@gmail.com (I.N.); karamabdelhai@gmail.com (K.A.E.H.); yoavarnson@gmail.com (Y.A.); yoram.neuman@clalit.org.il (Y.N.); ziyad.jabara@clalit.org.il (Z.J.); davidpe@tauex.tau.ac.il (D.P.); eddiekoman@gmail.com (E.K.); aassali@clalit.org.il (A.A.); hana100niki@gmail.com (H.V.-A.); 2 School of Medicine, Tel-Aviv University, Tel-Aviv 6997801, Israel; 3 Herzliya Medical Center, Herzliya 4685107, Israel; kobyp@hmc-ims.com

**Keywords:** aortic stenosis, transcatheter aortic valve implantation, on-site cardiac surgery, remote surgical backup

## Abstract

**Background**: Aortic stenosis (AS) is the most common valvular heart disease, associated with poor outcomes if left untreated. Current guidelines recommend that transcatheter aortic valve implantation (TAVI) procedures be performed in hospitals with an on-site cardiac surgery unit due to potential complications requiring surgical intervention. **Objective**: Based on our experience, we evaluated the feasibility and outcomes of implementing a TAVI program in a cardiology department without an on-site cardiac surgery unit, in collaboration with a remote hospital for surgical backup. **Methods**: The TAVI program involved pre- and post-procedural evaluations conducted at Meir Medical Center (Kfar Saba, Israel) with a remote surgical team available. The study population included 149 consecutive patients with severe aortic stenosis treated at the Meir valve clinic between November 2019 and December 2023. Procedures were performed by the center’s interventional cardiology team. **Results**: The mean age of the 149 patients was 80 ± 6 years, and 75 (50%) were female. The average STS score was 4.3, and the EuroSCORE II was 3.1. Among the patients, 68 (45%) were classified as New York Heart Association (NYHA) class III-IV. The valve types used included ACURATE neo2 (57 patients, 38%), Edwards SAPIEN 3 (43 patients, 28%), Evolut-PRO (41 patients, 27%), and Navitor (7 patients, 4%). There were no cases of moderate to severe paravalvular leak and no elevated post-implantation gradients, and there was no need for urgent cardiac surgery. One case of valve embolization was successfully managed percutaneously during the procedure. In-hospital follow-up revealed no deaths and only one major vascular complication. At one-year follow-up, six patients had died, with only one death attributed to cardiac causes. **Conclusions**: Our findings support the safe and effective performance of transfemoral TAVI in cardiology departments without on-site cardiac surgery, in collaboration with a remote surgical team. Further prospective, multicenter studies are warranted to confirm these results and guide broader clinical implementation of this practice.

## 1. Introduction

Aortic stenosis is a common yet serious valve disease, leading to significant morbidity and mortality if left untreated. Since transcatheter aortic valve implantation (TAVI) was introduced in 2002, the management of valvular heart disease has been revolutionized. The development of TAVI offers a less invasive option than traditional surgery and has demonstrated good outcomes for patients globally. The procedure has continuously evolved, with improvements leading to simplification and a significant reduction in complication rates according to recent studies [1,2,3]. As TAVI has evolved, efforts have shifted toward making the procedure more accessible, particularly in community and non-tertiary centers. One notable advancement is the move toward early discharge, with some patients even leaving the hospital on the same day as the procedure [4].

Despite its beneficial value, TAVI still carries a potential risk for major complications such as annular rupture, coronary artery obstruction, valve embolization, cardiac tamponade, and aortic dissection. However, the incidence of these complications remains low, ranging from 0.9 to 1.6% according to various studies [5,6]. The rising demand for TAVI has resulted in prolonged waiting times, which in turn leads to an increase in mortality, hospitalizations for heart failure, and urgent procedures. The mortality rate of AS patients awaiting TAVI ranges from 2% to 10% [7,8]. Given the rising demand for TAVI and the associated morbidity and mortality among patients on waiting lists, there is a need to enhance the capacity of experienced teams to treat this population. Current guidelines [9] recommend that TAVI procedures be performed at centers with on-site cardiovascular surgical capabilities, based on the assumption that surgical backup is beneficial in case of complications requiring emergency cardiac surgery (CS). Although catastrophic complications are rare (<0.7%), they can be life-threatening, with a 30-day mortality rate of 45% to 70% [10]. A meta-analysis of 9251 patients collected during the first decade of TAVI procedures found that the incidence of emergency CS during transarterial TAVI was around 1.1 ± 1.1% [11]. The evolution of transfemoral TAVI has been marked by significant advancements, including the development of new-generation valves, improved procedural planning, better complication prediction, and refined implantation techniques. These improvements have made the procedure much safer. Recent data show that the incidence of emergency CS is extremely low, at 0.4% to 0.7% [1,2]. These findings support the feasibility of performing selected TAVI procedures without on-site cardiac surgery, potentially reducing waiting time risks. The goal of this study is to evaluate the feasibility, safety, and outcomes of performing a TAVI program in a cardiology department without an on-site CS department, in collaboration with a remote hospital that has surgical backup.

## 2. Methods

### 2.1. Study Selection, Data Abstraction, and Validity Assessment and Analysis

This study included consecutive aortic stenosis patients treated at the valve clinic of Meir Medical Center (Kfar Saba, Israel) who underwent TAVI between November 2019 and December 2023. All patients were evaluated by a multidisciplinary heart team, including an interventional cardiologist, cardiothoracic surgeon, and cardiac imaging specialist, to determine their eligibility for TAVI. The assessments included clinical evaluations; complete echocardiography; calculation of the aortic valve area using the continuity equation; measurement of the left ventricular ejection fraction using both visual estimation and Simpson’s biplane method; and ECG-gated computerized tomography (CT) scans for annular measurements, calcium scores, and vascular access evaluations, in accordance with current guidelines.

The procedure was performed in a hospital affiliated with our healthcare organization (Clalit Health System). This facility is equipped solely with a cardiac operating room and lacks a dedicated cardiac surgery department and specialized surgical staff. TAVI procedures were conducted with cardiac surgical backup provided by a remote team, which was available to arrive at the hospital if needed. This remote backup team was notified prior to each case and remained on call throughout the procedure. Communication with the on-call team was maintained by phone and a secure digital link.

A dedicated vascular surgery department was available at Meir Medical Center and was consulted in cases involving access-related complications. The availability of vascular expertise was an integral part of the program’s design to ensure procedural safety. The procedures were conducted by experienced operators and staff using transfemoral access under local anesthesia and mild sedation. Device selection and sizing were determined by the heart team based on multidetector computed tomography (MDCT) and echocardiography results. A successful TAVI procedure was defined by the Valve Academic Research Consortium 2 (VARC 2) criteria [12], including correct valve placement, proper valve function, and the absence of major complications.

Patients were monitored post-procedurally at our hospital, and those with a stable condition were discharged following routine follow-up protocols. Data were collected on baseline characteristics, procedural outcomes, immediate post-operative results, complications, and 1-year mortality. Follow-up visits were conducted at the TAVI outpatient clinic one month after the procedure. Data on 30-day outcomes were gathered from follow-up visits and rehospitalization records.

Balloon-expandable transcatheter heart valves (THV) included the Sapien S3 valve (Edwards Lifesciences, Irvine, CA, USA). Self-expanding THV included the Evolut Pro+ (Medtronic, Galway, Ireland), Navitor (Abbott, Santa Clara, CA, USA) and ACURATE neo 2 (Boston Scientific, Marlborough, MA, USA). Vascular closure devices included the Perclose Prostyle and Perclose Proglide (Abbott, Santa Clara, CA, USA), MANTA (Teleflex Inc. Wayne, PA, USA) and Angio-Seal (Terumo, Somerset, New Jersey, USA).

### 2.2. Study Endpoints

The study’s endpoints were based on the Valve Academic Research Consortium-2 (VARC-2) criteria [12]. The primary endpoint was device success, defined by the absence of procedural mortality, accurate valve placement, and the absence of significant valve gradients or regurgitation. Secondary endpoints included cardiovascular death, all-cause mortality, and early safety measures such as stroke, bleeding, and vascular complications at 30 days and 1 year. Valve safety was assessed by evaluating valve degeneration, valve dysfunction, endocarditis, thrombosis, thromboembolic events, and bleeding events related to valve therapy.

### 2.3. Statistical Analysis

A retrospective analysis was performed on the patients who underwent TAVI between November 2019 and December 2023. Demographic and clinical baseline characteristics, as well as procedural parameters, are presented as absolute numbers and percentages. Continuous variables are expressed as the mean and standard deviation or as the median and interquartile range. All statistical analyses were performed using IBM SPSS Statistics for Windows, version 28.0 (IBM Corp., Armonk, NY, USA). The study was approved by the Meir Medical Center Ethics Committee (approval number 0032-23-MMC), with approval granted from 2019 through 2025. All procedures were conducted in accordance with the Declaration of Helsinki.

## 3. Results

These outcomes were assessed to evaluate the feasibility and safety of performing TAVI in the absence of on-site surgical support. The baseline characteristics are detailed in Table 1. The study population consisted of 149 patients with a mean age of 80.5 ± 6.4 years, of which 50% were female. The mean STS score was 4.3 ± 2.9, the mean EuroSCORE II was 3.1 ± 2.2, and 45.6% of patients were in NYHA functional class III-IV. A significant proportion (55%) of patients had a history of coronary artery disease (CAD), 28% had chronic kidney disease (CKD), and 17% had peripheral vascular disease (PVD). About half (45%) of the study population had diabetes, a quarter (25%) had atrial fibrillation (AF), and the minority (7%) had a permanent pacemaker before the procedure.

**Figure 1:**
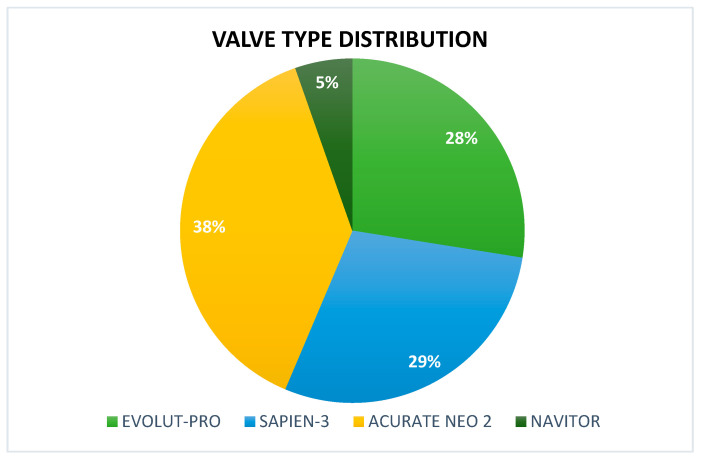
Valve types implanted in this study. Sapien S3 valve (Edwards Lifesciences, Irvine, CA, USA). Evolut Pro+ (Medtronic, Galway, Ireland), Navitor (Abbott, Santa Clara, CA, USA) and ACURATE neo 2 (Boston Scientific, Marlborough, MA, USA).

In the pre-procedural echocardiographic assessment (Table 1), moderate to severe mitral regurgitation was observed in eight patients (5.4%), and moderate to severe tricuspid regurgitation were identified in one patient (0.7%) in addition to severe aortic stenosis. The mean aortic valve gradient was 46.9 ± 15.5 mmHg, the mean calculated aortic valve area was 0.75 ± 0.15 cm^2^, and the mean peak aortic velocity was 4.59 ± 0.55 m/s. The pre-procedural CT Analysis showed a mean calcium score of 2065 ± 610 and a mean Annular Perimeter of 76.8 ± 6.5. All TAVI procedures were carried out under local anesthesia and mild sedation. All procedures were performed with trans-femoral access. Among the valve types used in this study, ACURATE neo2 was the most common (38%), followed by Edwards SAPIEN 3 (29%), Evolut-PRO (28%), and Navitor (5%) (Figure 1).

Few (4.7%) patients received TAVI in a bioprosthetic valve (Table 2). Hemostasis for the large-bore access site was predominantly achieved using Prostyle and Angioseal (57%), and access to the rest of the sites was achieved with Prostyle alone (14%) or Manta (28%). During the procedure, no cases of emergency surgery, annular rupture, coronary obstruction, or cardiac tamponade were observed (Table 2). We had one case of valve embolization to the thoracic aorta that was resolved percutaneously with implantation of another valve in the aortic position.

**Figure 2:**
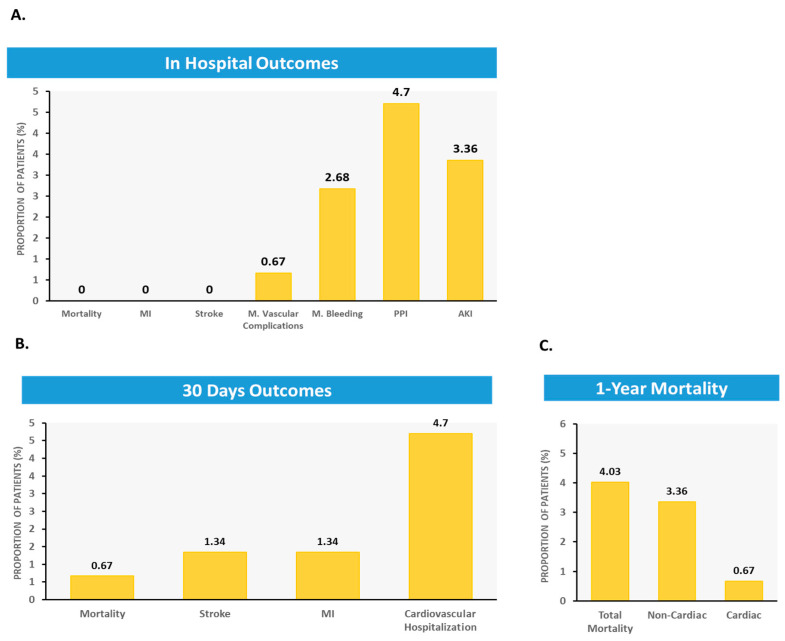
(**A**) In-hospital outcome, during the hospitalization of the index procedure with a median of 2 days (**B**) Evaluated 30-days outcomes (**C**) Cardiac and non-cardiac mortality at one year. All data represents a cohort of 149 patients, undergoing procedure between 2019–2023 at Meir hospital center. MI = Myocardial Infarction; AKI = Acute Kidney Disease; PPI = permanent pacemaker implantation.

The average discharge time was 2.1 ± 1.7 days, with the majority of patients discharged within a median time of two days. Clinical outcomes are detailed in Table 3 and Figure 2. 

The no. of cases of in-hospital mortality, periprocedural MI, and stroke were reported during the study period. We identified one case (0.6%) of a major vascular event, specifically a pseudoaneurysm in the groin, complicated by significant bleeding. This was managed conservatively without surgical intervention. Additionally, there were two cases of major bleeding events: one involved upper gastrointestinal bleeding treated endoscopically with a blood transfusion, and the other was a hematoma in the groin managed conservatively with a blood transfusion. In total, there were three cases (2%) of major bleeding events observed, and there were no instances of mortality among these patients during the one-year follow-up period. Seven (4.7%) patients required the implantation of a new pacemaker. The mean post-procedural aortic gradient was 11 ± 6 mmHg, and no cases of significant peri-valvular leak (PVL) were observed in post-procedural echocardiography (Table 3).

During the first 30 days following the procedure, one patient died from a non-cardiac cause, two patients (1.3%) had a stroke, and seven patients (4.7%) were hospitalized due to a cardiovascular cause (Table 3). Six patients died during the first year of follow-up, with only one dying due to a cardiac cause. Among the non-cardiac deaths, one patient died from pneumonia, one from pancreatic cancer, another from sepsis, a fourth from renal disease, and another from an unknown cause (Table 3).

## 4. Discussion

In this study, we examined a cohort of 149 patients with symptomatic severe aortic valve stenosis who underwent transfemoral TAVI, following a structured program. This program included heart team discussions, TAVI procedures performed at a center without a cardiac surgical unit on-site but with remote surgical backup. Our study demonstrated encouraging outcomes, with no cases of emergency cardiac surgery, low complication rates, and successful procedural characteristics. We observed no instances of moderate to severe paravalvular leaks, only one major vascular complication, and no in-hospital mortality. During the entire follow-up period, only one patient died from a cardiac-related cause. These results highlight the efficacy and safety of our TAVI program, even in the absence of an on-site cardiac surgery department.

Despite the increasing maturity of TAVI, it remains a procedure with the potential for life-threatening complications [13]. Earlier studies conducted about a decade ago reported the need for emergent cardiac surgery in 1–5% of cases [14,15], whereas more recent studies show this requirement in less than 1% of cases [1,2,16]. This rate is expected to decline further with advancements in operator experience, procedural planning, and the development of repositionable TAVI systems and improved valve designs. In our study, no cases required conversion to cardiac surgery during or after the procedure.

A large German study (AQUA) [16] compared the outcomes of transfemoral TAVI at hospitals with and without on-site cardiac surgery departments. Patients undergoing TAVI at hospitals without cardiac surgery were older and had more comorbidities. However, the incidence of severe intra-procedural complications (such as annular rupture, aortic dissection, coronary obstruction, and device embolization) was rare (<1%) and actually lower in patients treated at hospitals without on-site cardiac surgery departments. In a separate analysis by Eggebrecht et al. [16] of more than 1000 TAVI patients treated at hospitals without on-site cardiac surgery, it was shown that the heart teams at these non-CS sites selected patients similarly to those at CS sites. Procedural outcomes regarding in-hospital complications and mortality did not differ statistically between institutions with and without on-site cardiac surgery departments. In accordance with those studies and with our results, a recent Meta-analysis showed that outcomes of patients undergoing TAVI at institutions without on-site CS are similar to those of patients treated at centers with a CS department on-site [17].

In our study, there were no in-hospital deaths or major complications. However, as mentioned, one major procedural complication with a potential need for urgent surgery was observed and treated percutaneously during the index procedure. This underscores the importance of highly experienced and skilled TAVI teams, especially when on-site cardiac surgery is unavailable.

Over time, there has been a consistent reduction in the length of hospital stays following TAVI, regardless of the patient’s surgical risk. Early-discharge protocols, such as those outlined in the Vancouver 3M and FAST-TAVI registries, are expected to further reduce hospital stays following TAVI [18,19]. The (R-) EXPRESS program has demonstrated the feasibility of early discharge, either to the patient’s home or a referral hospital, while maintaining safety and optimizing TAVI programs. This program reported a median length of stay of two days [20]. Similarly, our study revealed a median length of stay of two days.

Nowadays, the complication rate mandating urgent surgery is lower than 0.5%. Therefore, we believe that the presence of on-site CS is not the primary safety concern [17]. Instead, the critical factors include thorough procedure planning, pre- and post-operative evaluations, and collaboration among the heart team. TAVI programs without on-site cardiac surgery should be carried out by experienced centers with teams familiar with all stages of planning and performing the procedure and post-procedural care, including prompt recognition of post-operative cardiac and non-cardiac complications. When these conditions are met, the procedure can be performed safely, even without on-site CS. Implementing TAVI in centers without on-site cardiac surgery presents unique challenges but also opportunities for innovation in patient care. Our experience demonstrated that, with careful planning and collaboration, these challenges can be overcome, leading to outcomes comparable to those seen in centers with cardiac surgery departments. These findings are particularly valuable for healthcare systems seeking to broaden access to TAVI in regions with limited surgical infrastructure. Establishing remote surgical standby protocols may serve as a pragmatic and scalable strategy to safely expand TAVI access.

### Study Limitations

Our study has several notable limitations. First, there is potential selection bias due to the early phase of our TAVI program. As this program was newly established at our hospital, the initial cases involved relatively uncomplicated patients to ensure procedural safety and optimize outcomes. However, we did not exclude any patients based on risk profile, and this study presents the first 150 consecutive patients treated at our center. Future studies with a broader and more complex patient population will be necessary to further validate our findings.

Additionally, this is a single-center, retrospective, non-randomized observational study, which may introduce inherent methodological biases. The outcomes are highly dependent on the expertise and experience of the operators, which limits the generalizability of the findings to other centers. The distribution of different valve types used for TAVI could also have influenced the results, and we were unable to assess the impact of valve selection on major complications, post-implantation paravalvular leak, or pacemaker rates. Lastly, our analysis is limited to evaluating in-hospital and 1-year outcomes, with no long-term follow-up data available.

## 5. Conclusions

The case studies presented in this paper demonstrate favorable outcomes for patients undergoing transfemoral TAVI in institutions without an on-site cardiac surgery department, with strong safety profiles and without heightened risk to patients. Our successful TAVI program highlights the potential for similar models in other institutions. We emphasize the need for prospective, multicenter, randomized trials to further validate the safety and feasibility of this approach.

## Figures and Tables

**Table 1:** Characteristics of the patients at baseline.

Variable	N = 149
Age (y) (mean ± SD)	80.5 ± 6.4
Female, no. (%)	75 (50.3%)
Body-mass index (mean ± SD)	28 ± 7.1
Time from diagnosis to TAVI (mean ± SD)	55 days ± 9
STS score (mean ± SD)	4.36 ± 2.96
EuroSCORE II score (mean ± SD)	3.16 ± 2.27
NYHA class III/IV, no. (%)	68 (45.6%)
Previous myocardial infarction, no. (%) (N = 131)	15 (11.4%)
Coronary artery disease, no. (%)	83 (55.7%)
Prior PCI, no. (%)	50 (33.5%)
Peripheral vascular disease (%)	26 (17.45%)
Previous valvular surgery, no. (%)	9 (6%)
Hypertension, no. (%)	125 (84%)
Previous stroke, no. (%)	20 (13.4%)
Diabetes Mellitus, no. (%)	68 (45.6%)
Hyperlipidemia, no. (%)	130 (87.2%)
Atrial fibrillation, no. (%)	38 (25.5%)
COPD, no. (%)	25 (16.8%)
CKD (eGFR < 60 mL/min/1.73 m^2^), no. (%)	41 (27.5%)
Anemia (Hg < 10 mg/dL), no.	76 (51%)
Permanent pacemaker, no. (%) (N = 134)	10 (7.4%)
Echocardiographic data	
Aortic valve area (cm^2^)	0.75 ± 0.15
Max aortic valve gradient (mm Hg) (mean ± SD)	75 ± 23.3
Mean aortic gradient (mm Hg) (mean ± SD)	46.9 ± 15.5
Peak aortic valve velocity (m/s) (mean ± SD)	4.59 ± 0.55
LVEF, % (mean ± SD)	57.8 ± 9.9
Moderate to severe mitral regurgitation	8 (5.4%)
Moderate to severe tricuspid regurgitation	1 (0.7%)
CT analysis	
Calcium, score (mean ± SD), annular	2065 ± 610
Annular Perimeter (mean ± SD)	76.85 ± 6.57

Values are N (%) or mean ± SD. STS = Society of Thoracic Surgeons risk score; NYHA = New York Heart Association; PCI = percutaneous coronary intervention; COPD = chronic obstructive pulmonary disease; CKD = chronic kidney disease; CT = computed tomography.

**Table 2:** Procedural characteristics and intra-procedural complications.

Variables	N = 149
Prosthesis type	no. (%)
Evolut-PRO	41 (27.5%)
Edwards SAPIEN 3	43 (28.8%)
ACURATE neo2	57 (38.2%)
Navitor	8 (5.4%)
Valve Platform (N)	Size Distribution (%)
Evolut-PRO (n = 41)	S (40%), M (50%), L (10%)
Edwards SAPIEN 3 (n = 43)	23 mm (30%), 26 mm (45%), 29 mm (25%)
ACURATE neo2 (n = 57)	26 mm (20%), 29 mm (55%), 34 mm (25%)
Navitor (n = 8)	23 mm (37.5%), 25 mm (37.5%), 27 mm (25.0%)
Valve in bioprosthetic valve (%)	7 (4.7%)
Closure device (valve access only) (%)	
Prostar/Proglide	21 (14.1%)
Prostar/Proglide + Angioseal	86 (57.7%)
Manta	42 (28.2%)
Intra-procedural complications	
Conversion to surgery	0 (0%)
Emergent surgery	0 (0%)
Need for a second valve	1 (0.67%)
Coronary obstruction	0 (0%)
Cardiac tamponade	0 (0%)
Annular rupture	0 (0%)
Valve migration/embolization	1 (0.67%)

Values are N (%).

**Table 3:** In-hospital, 30-day, and 1-year outcomes.

In-hospital outcomes	N = 149
In-hospital death	0 (0%)
Peri-procedural MI	0 (0%)
Stroke	0 (0%)
Major vascular complication	1 (0.67%)
Major bleeding (Type 2 ≥ BARC 3)	4 (2.68%)
Permanent pacemaker implantation	7(4.7%)
Acute renal failure	5 (3.36%)
Hemodialysis	0 (0%)
Time to discharge (days, mean ± SD)	2.1 ± 1.7
Echocardiographic findings—in-hospital post-procedural evaluation	
Ejection fraction, % (mean ± SD)	60 ± 8
mean aortic valve gradient, mm Hg (mean ± SD)	11.25 ± 6.2
Moderate or severe AR	0 (0%)
30-day outcomes	
Mortality	1/149 (0.67%)
Myocardial Infarction	2/149 (1.34%)
Stroke	2/149 (1.34%)
Cardiovascular hospitalization	7/149 (4.7%)
HALT	1 (0.67%)
Endocarditis	1 (0.67%)
Acute coronary syndrome	2 (1.34%)
Heart failure	3 (2%)
Non-cardiovascular hospitalization	14 (9.39%)
Mortality—1 year	
Total	6/149 (4.0%)
Non-cardiac	5/149 (3.36%)
Cardiac	1/149 (0.67%)

Values are N (%) or mean ± SD.

## Data Availability

The original contributions presented in this study are included in the article. Further inquiries can be directed to the corresponding author.
